# Supplementary material for: Global climate-driven trade-offs between the water retention and cooling benefits of urban greening
Source: Nat Commun. 2022 Jan 26;13:518. doi: 10.1038/s41467-022-28160-8 (PMC8792007; doi:10.1038/s41467-022-28160-8)
Supplement: Supplementary file 1 — Supplementary Information [file 41467_2022_28160_MOESM1_ESM.pdf]

Supplementary Information for:

Global climate-driven trade-offs between the water retention and cooling benefits of urban greening

Authors: M. O. Cuthbert[1,2]*, G. C. Rau[3], M. Ekström[1], D. M O'Carroll[2], A. J. Bates[4].

Affiliations:

[1]School of Earth and Environmental Sciences, Cardiff University, Cardiff, UK.

[2]School of Civil and Environmental Engineering, The University of New South Wales, Sydney, Australia.

[3]Institute of Applied Geosciences, Karlsruhe Institute of Technology, Karlsruhe, Germany.

[4]School of Animal, Rural & Environmental Sciences, Nottingham Trent University, Nottingham, UK.

*Correspondence to: cuthbertm2@cardiff.ac.uk

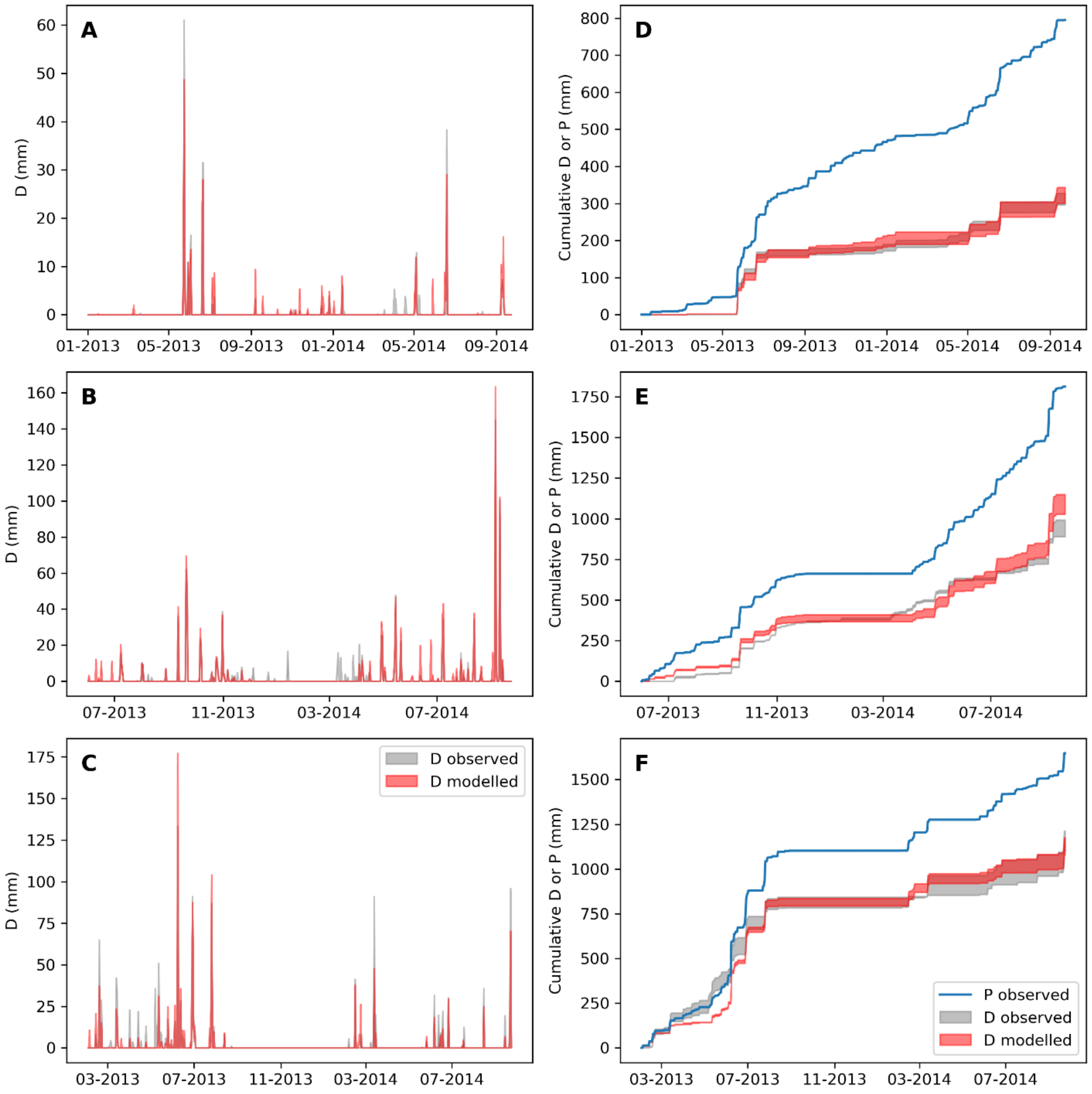

Supplementary Figure 1. Time series of modelled and observed green roof drainage (D) and cumulative precipitation (P) and drainage for behavioral simulations for 3 Canadian roofs in different climates located in (**A,D**) Halifax, (**B,E**) Calgary and (**C,F**) London.

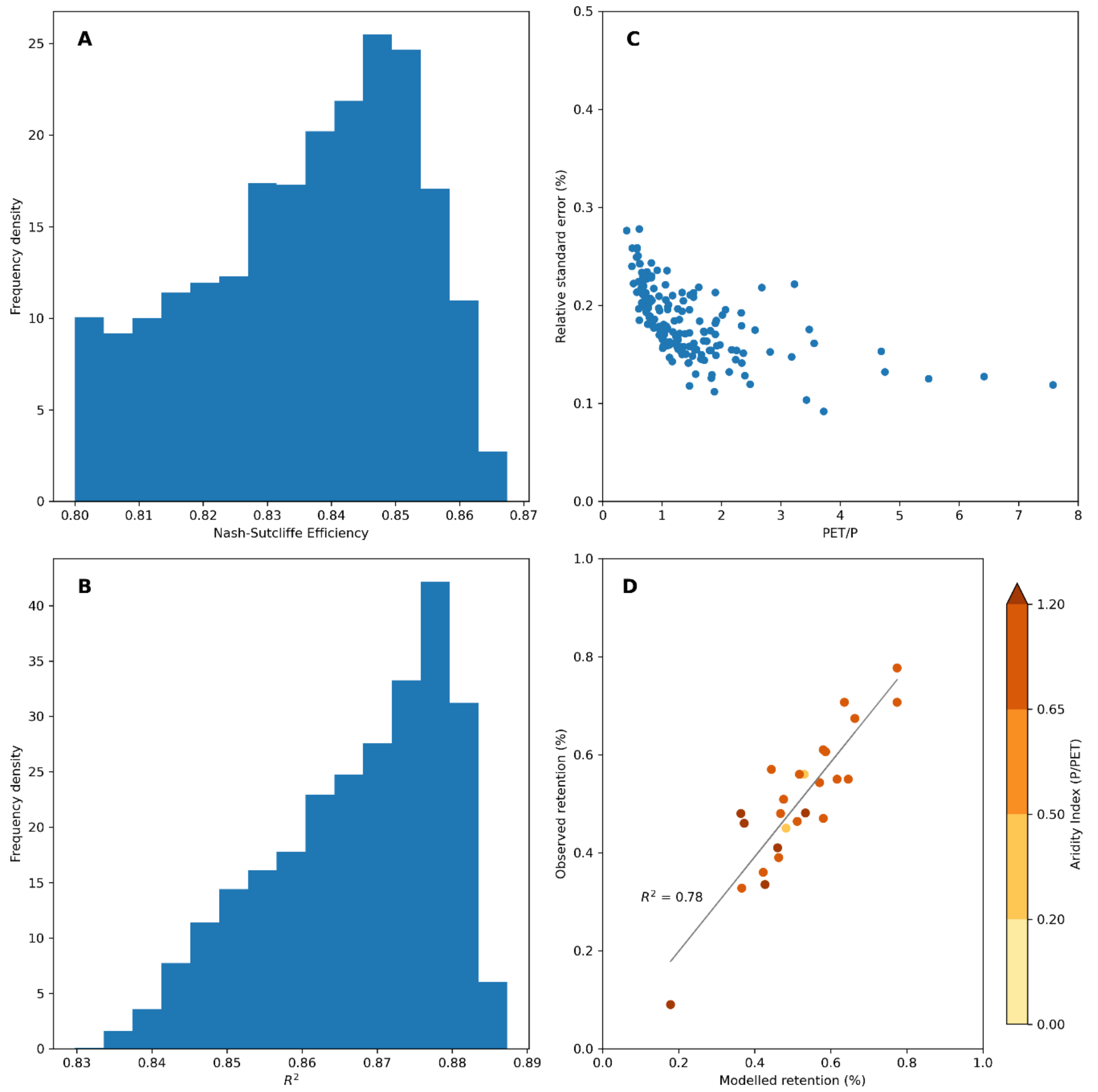

Supplementary Figure 2. Model evaluation statistics for best performing Monte-Carlo Experiments (MCEs) expressed as means for simulations of 3 Canadian roofs in different climates (**A**) Nash-Sutcliffe Efficiency in modelled versus observed drainage (**B**) Coefficient of determination in modelled versus observed drainage. (**C**) Relative standard error for the outputs of all behavioral parameter sets derived from the MCEs applied to the collated GSOD city locations, plotted against aridity. (**D**) Observed versus modelled mean retention for 26 green roofs from 13 published studies.

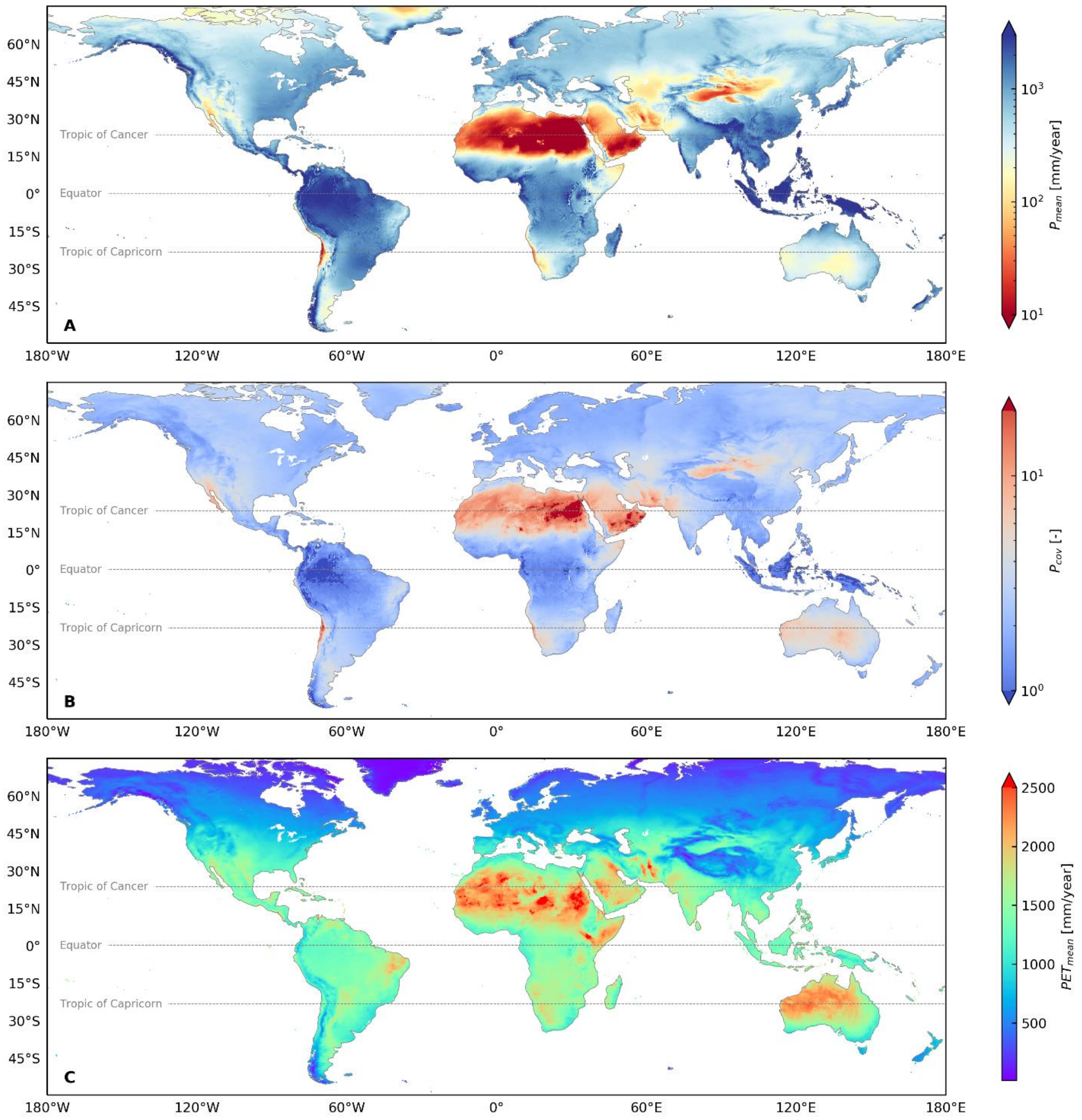

Supplementary Figure 3 Maps of ERA5 derived global climate parameters for (**A**) mean precipitation (P) (**B**) coefficient of variation in daily precipitation (P_{cov}) and (**C**) mean reference crop potential evapotranspiration (PET)

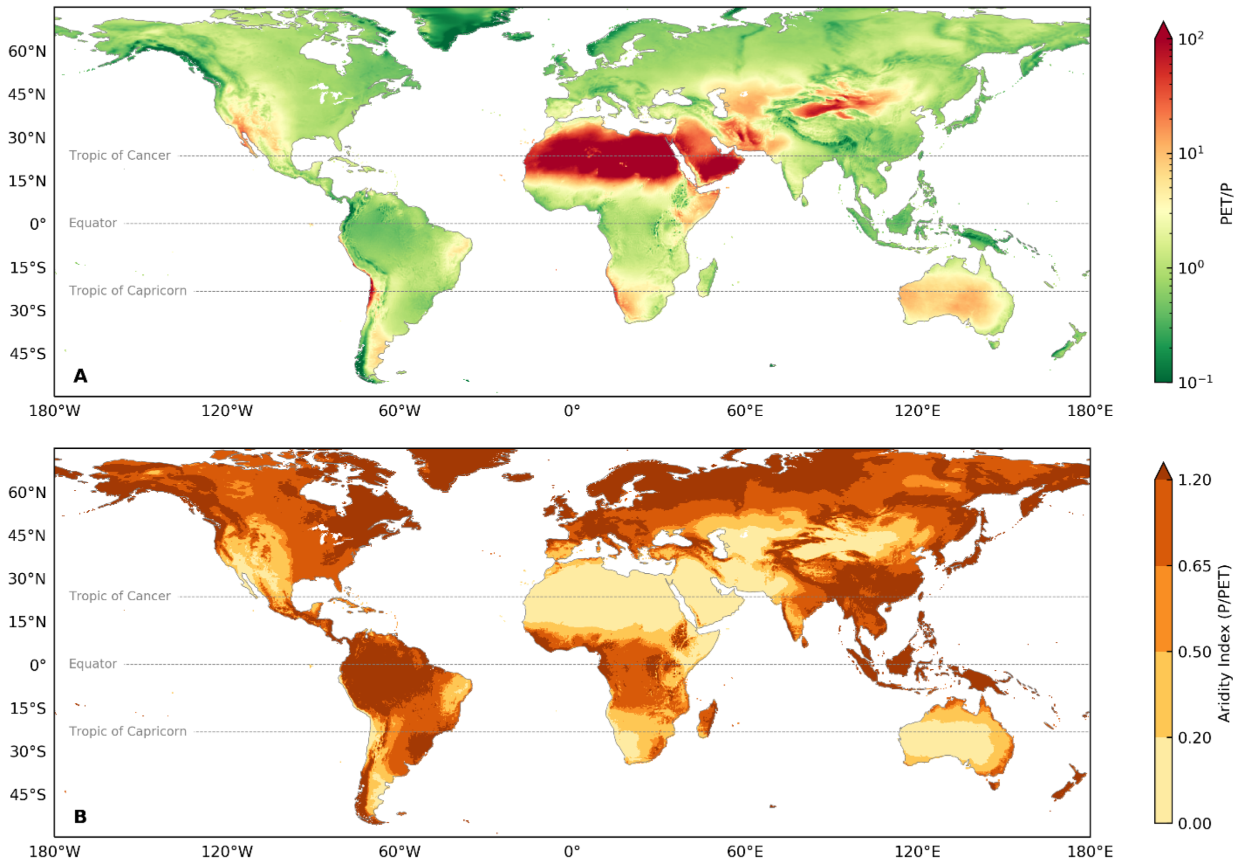

Supplementary Figure 4. Maps of ERA5 derived global climate parameters for (**A**) mean reference crop potential evapotranspiration divided by mean precipitation (**B**) Aridity Index equal to mean precipitation divided by mean reference crop potential evapotranspiration.

.

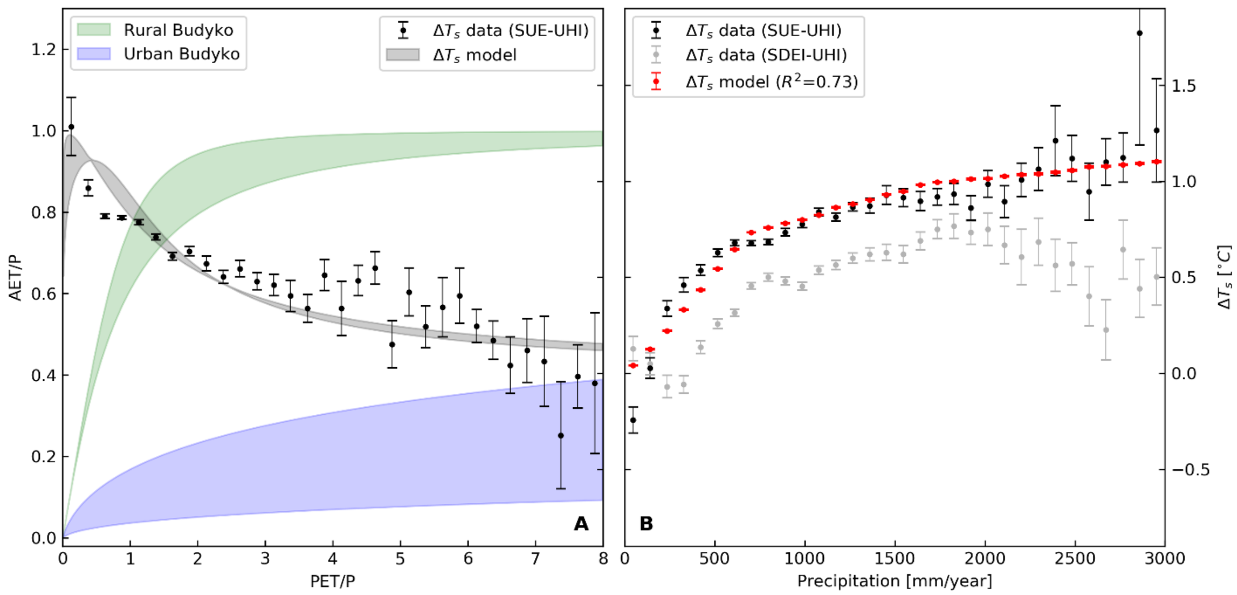

Supplementary Figure 5. (**A**) Comparison of mean urban-rural surface temperature difference (SUE-UHI[21] ΔTs) with the empirical model given by Equation 15 across a range of realistic urban-to-rural Budyko curves. (**B**) Evaluation of best model fit against Precipitation. SDEI-UHI data[20] also shown for comparison. Error bars are equal to the SEM.

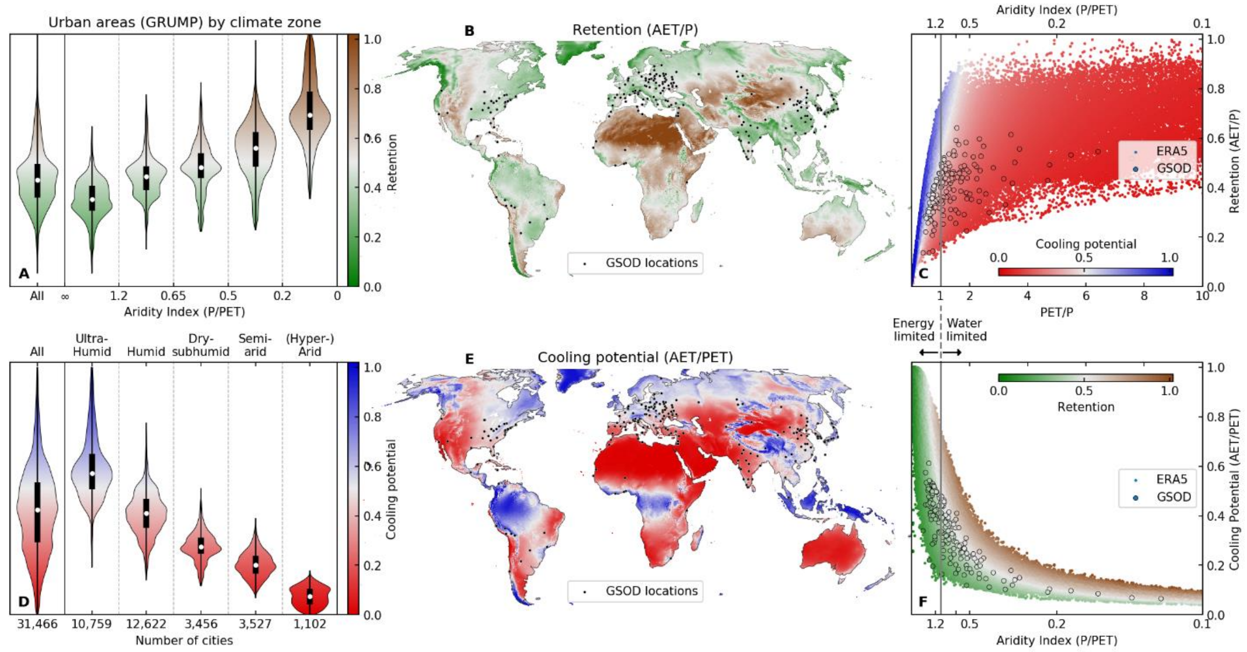

Supplementary Figure 6. Global distributions and interrelationships of ERA5 forced models using 'extensive substrate' (h = 50 mm) for: (**B, C**) Retention and (**E, F**) Cooling Potential. (**A, D**) Violin plots extracted for GRUMP urban areas only. GSOD city point-data and locations shown for reference in (**B, C**) and (**E, F**).

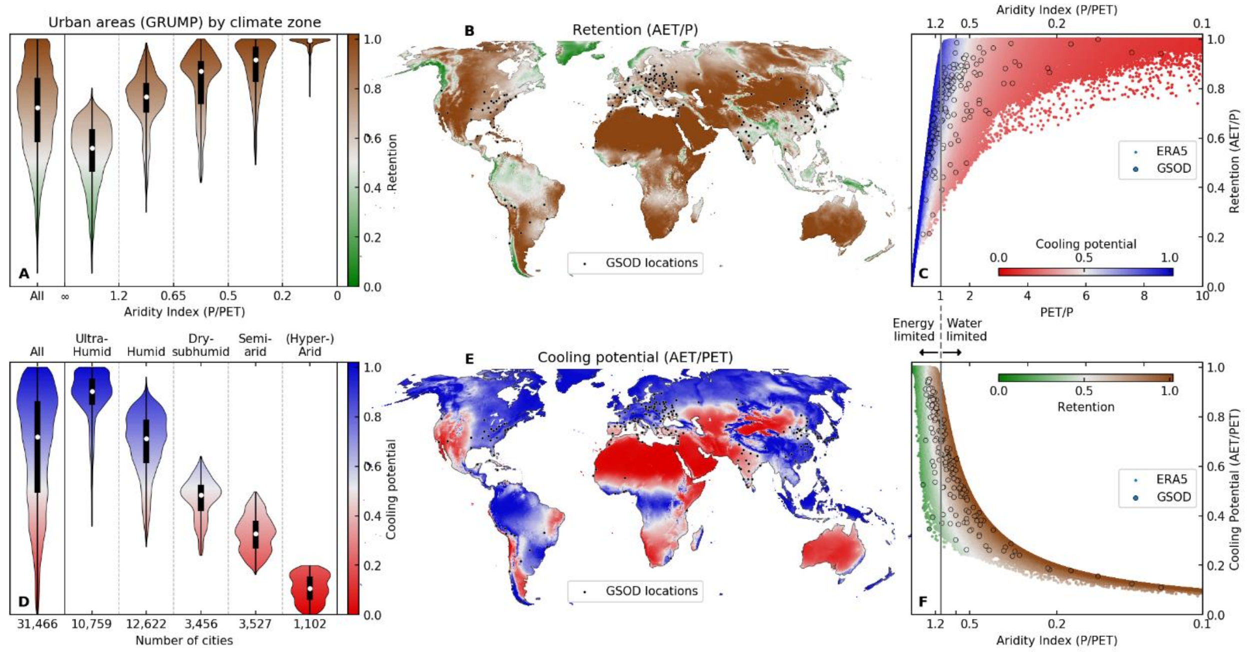

Supplementary Figure 7. Global distributions and interrelationships of ERA5 forced models using 'deep substrate' (h = 1000 mm) for: (**B,C**) Retention and (**E,F**) Cooling Potential. (**A**,**D**) Violin plots extracted for GRUMP urban areas only. GSOD city point-data and locations shown for reference in (**B, C**) and (**E, F**).

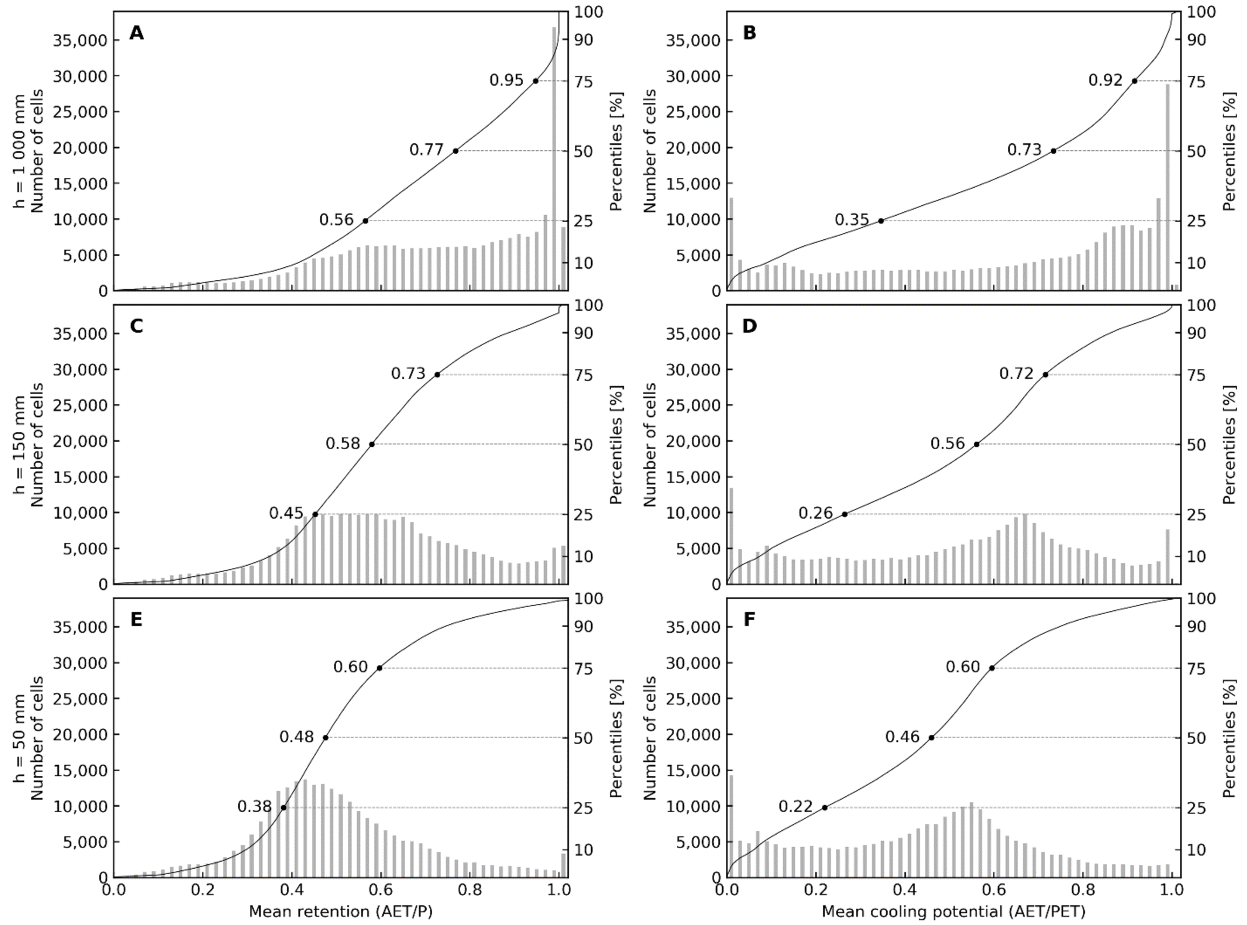

Supplementary Figure 8. Histograms of global mean retention and cooling potential for ERA5 forced simulations for (**A,B**) deep substrate (h = 1000 mm) (**C,D**) intensive substrate (h = 150 mm) (**E,F**) extensive substrate (h = 50 mm)

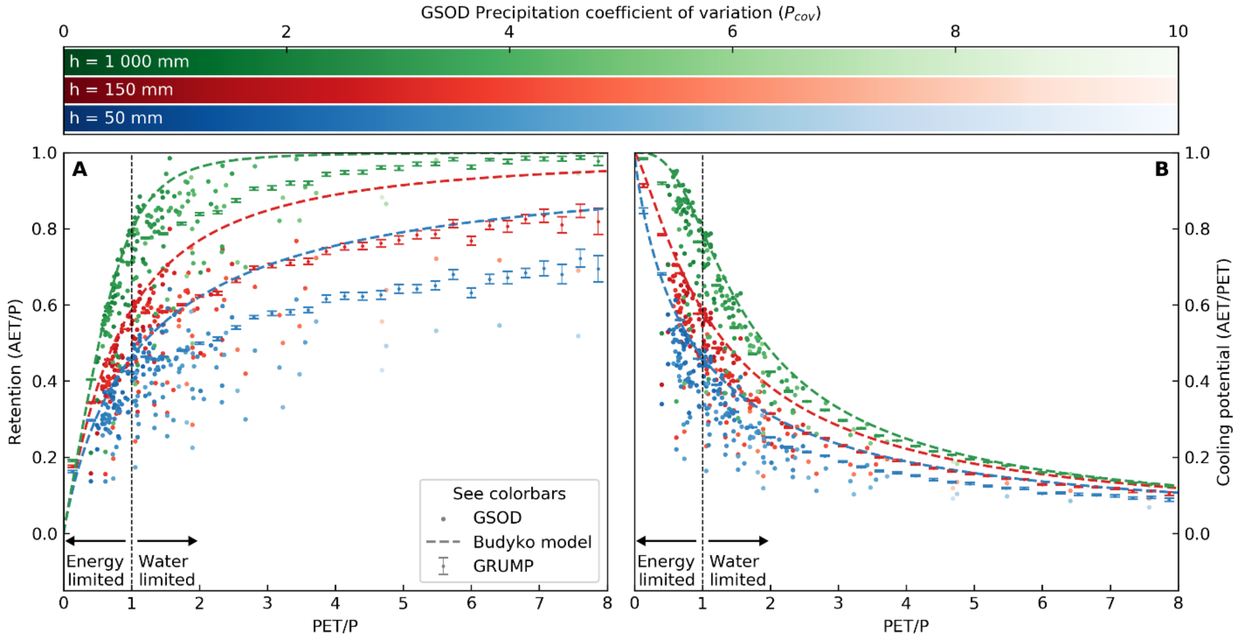

Supplementary Figure 9. Comparisons between modelled (**A**) retention and (**B**) cooling for GSOD and GRUMP locations. Upper bounding Budyko curves lambda values are 3, 1.3 and 0.9 for deep (h = 1000 mm), intensive (h = 150 mm), and extensive (h = 50 mm), substrate depths respectively. GRUMP values are mean values with error bars as SEM.

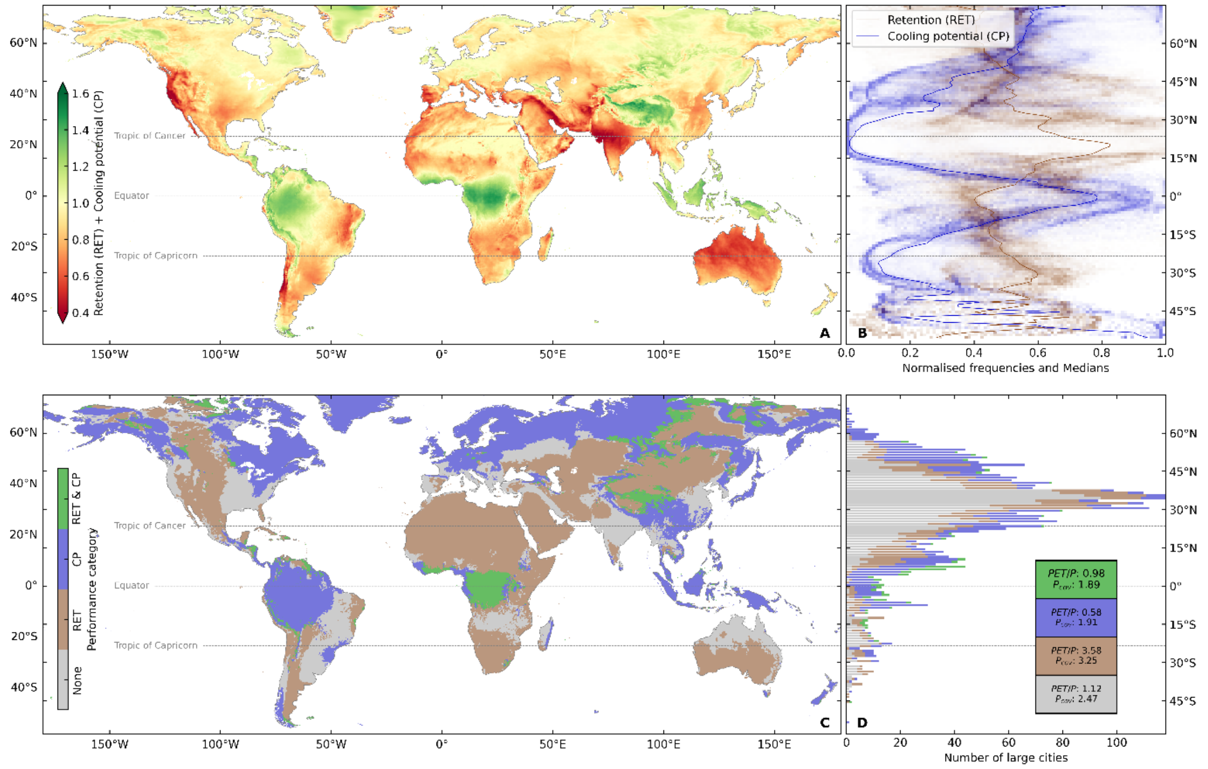

Supplementary Figure 10. Global trade-offs between mean retention and cooling potential. (A) Global map of the sum of mean retention and cooling potential metrics. (B) Latitudinal summaries of mean retention and cooling potential. Shading maps the binned frequencies normalized for 1° latitude bands. Solid lines are medians across the latitudes. (C) Categorized performance of mean retention only >0.5 (RET), mean cooling potential only >0.5 (CP) mean retention and mean cooling potential both >0.5 (RET & CP) or neither mean retention or mean cooling potential >0.5 (None). (D) Latitudinal summaries by categories defined in (C) for large cities (defined as having population > 100 000 in the year 2000 CE). Inset indicates the median value of PET/P and coefficient of variation in daily precipitation (Pcov) for each mapped category. All results shown are for extensive substrates (h = 50 mm).

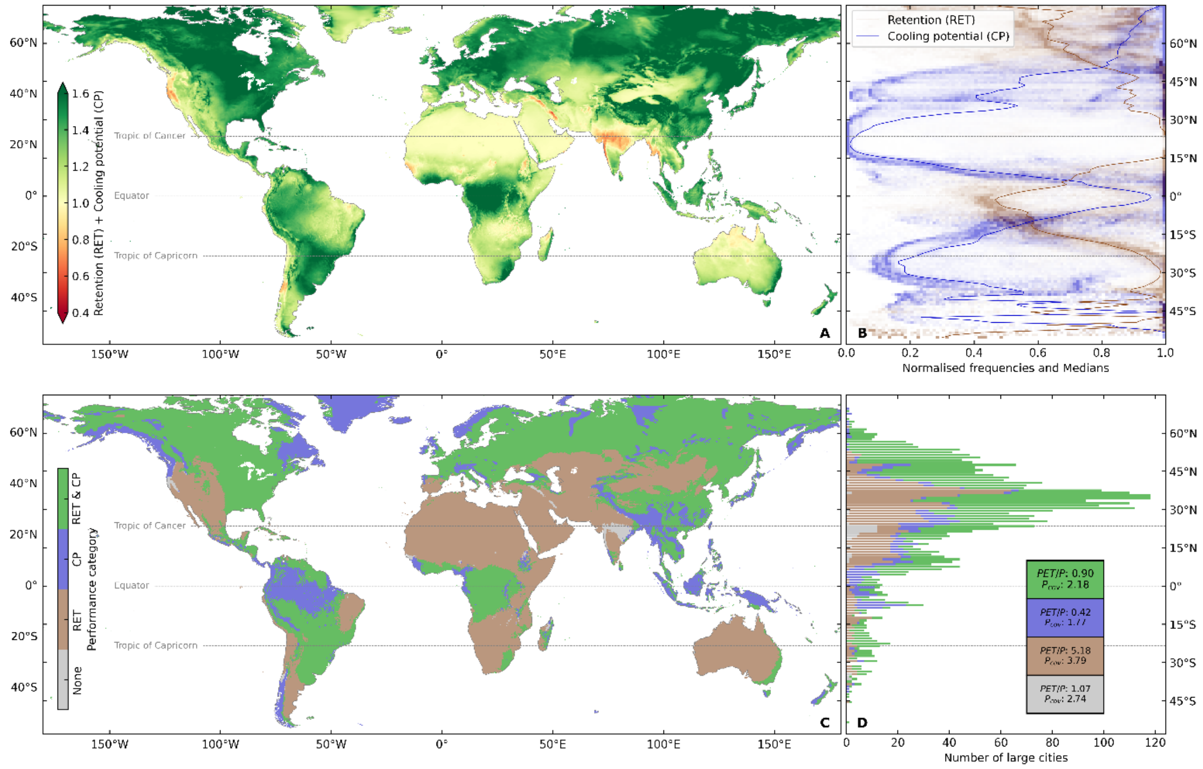

Supplementary Figure 11. Global trade-offs between mean retention and cooling potential. (A) Global map of the sum of mean retention and cooling potential metrics. (B) Latitudinal summaries of mean retention and cooling potential. Shading maps the binned frequencies normalized for 1° latitude bands. Solid lines are medians across the latitudes. (C) Categorized performance of mean retention only >0.5 (RET), mean cooling potential only >0.5 (CP) mean retention and mean cooling potential both >0.5 (RET & CP) or neither mean retention or mean cooling potential >0.5 (None). (D) Latitudinal summaries by categories defined in (C) for large cities (defined as having population > 100 000 in the year 2000 CE). Inset indicates the median value of PET/P and coefficient of variation in daily precipitation (Pcov) for each mapped category. All results shown are for deep substrates (h = 1000 mm).

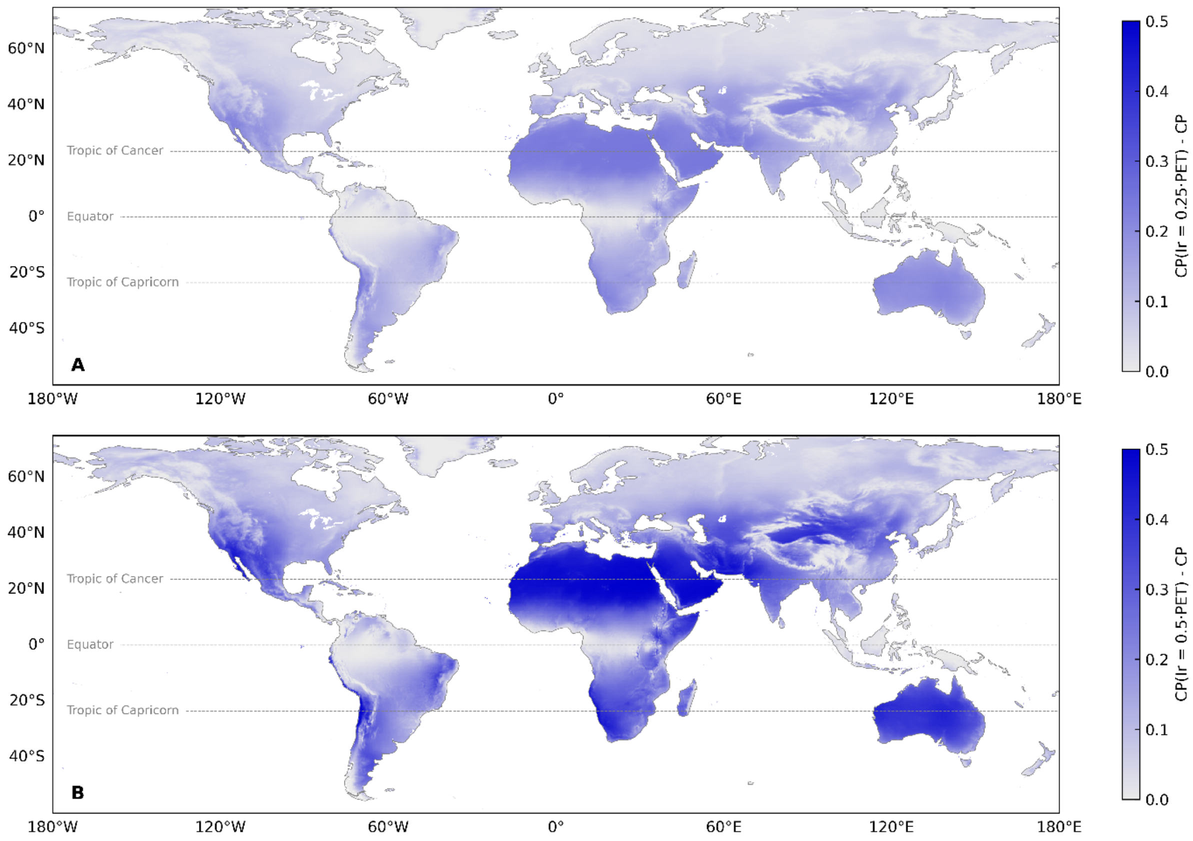

Supplementary Figure 12. Global distribution of the change in cooling potential (CP) due to irrigation (Ir) scenarios for (**A**) irrigation equal to 25% of PET on non-rain days (**B**) irrigation equal to 50% of PET on non-rain days
